# Demonstration of Parthenogenetic Reproduction in a Pet Ball Python (*Python regius*) through Analysis of Early-Stage Embryos

**DOI:** 10.3390/genes14091744

**Published:** 2023-08-31

**Authors:** Francesco Di Ianni, Sara Albarella, Alessandro Vetere, Marco Torcello, Michela Ablondi, Mariagiulia Pugliano, Susanna Di Mauro, Pietro Parma, Francesca Ciotola

**Affiliations:** 1 Department of Veterinary Science, Strada del Taglio 10, 43121 Parma, Italy; 2 Department of Veterinary Medicine and Animal Production, University of Naples Federico II, Via Delpino 1, 80137 Naples, Italy; 3 Ambulatorio Veterinario Dott. Di Mauro, Via Parini 8, 24043 Caravaggio, Italy; 4 Department of Agricultural and Environmental Sciences, Via Celoria 2, 20133 Milano, Italy

**Keywords:** parthenogenesis, *P. regius*, VNTR, PCR, genetic, reptiles

## Abstract

Parthenogenesis is an asexual form of reproduction, normally present in various animal and plant species, in which an embryo is generated from a single gamete. Currently, there are some species for which parthenogenesis is supposed but not confirmed, and the mechanisms that activate it are not well understood. A 10-year-old, wild-caught female ball python (*Python regius*) laid four eggs without any prior contact with a male. The eggs were not incubated and, after 3 days, were submitted to the University of Parma for analysis due to the suspicion of potential embryo presence. Examination of the egg content revealed residual blood vessels and a small red spot, indicative of an early-stage embryo. DNA was extracted from the three deceased embryos and from the mother’s blood, five microsatellites were analyzed to ascertain the origin of the embryos. The captive history data, together with the genetic microsatellite analysis approach, demonstrated the parthenogenetic origin of all three embryos. The embryos were homozygous for each of the maternal microsatellites, suggesting a terminal fusion automixis mode of development.

## 1. Introduction

Parthenogenesis (from the two Greek words *parthenos*, meaning virgin, and *genesis,* meaning origin) has been defined as a form of ‘asexual’ reproduction in which a female reproduces without the participation of a male [1,2,3,4,5]. The term ‘parthenogenesis’ was later defined by Richard Owen in 1849 as “procreation without the immediate influence of the male”. In fact, the embryo forms without male fertilization by sperm [6,7,8]. These embryos do not always develop into new individuals because they often suffer from high mortality; therefore, the definition of parthenogenesis does not consider the development of new individuals [1] but only of embryos [9]. Parthenogenetic events have been observed in both plants and animals [4,5]. Several mechanisms of parthenogenesis have been demonstrated. Meiosis occurs in automictic or meiotic parthenogenesis, but the mother’s chromosomal constitution is reestablished through various mechanisms. Some of these mechanisms result in homozygosity at all loci, while others transmit the mother’s genome intact to the offspring. On the other hand, in apomictic or mitotic parthenogenesis, eggs are formed through a series of mitotic divisions, effectively bypassing the process of meiosis [10]. Unlike asexual reproduction, in which new individuals are formed from somatic cells, parthenogenesis involves the development of egg cells with meiotic recombination of the genetic material coming from parents as an “incomplete form of sexual reproduction” [3,9]. In most cases, as there is no genetic recombination, the progeny is genetically identical to its virgin mother [11]. Since parthenogenesis can occur in different ways within the life cycles of different animals, three classifications have been proposed according to different criteria, namely reproduction, sex determination and cytological mechanisms [1,9]. In a broad sense, parthenogenetic lineages are typically classified as either ‘generalists’ or ‘specialists’, hinging on their possession of a broad ecological niche and adaptability to diverse environments, or their exceptional fitness within a constrained set of surroundings [5]. Parthenogenesis can be divided into (i) tychoparthenogenesis, when unfertilized eggs occasionally develop through parthenogenesis; (ii) facultative parthenogenesis, when the eggs may be fertilized or develop parthenogenetically and (iii) obligate parthenogenesis, when eggs always develop parthenogenetically. Among squamate reptiles, approximately 30 unisexual species have been formally named as belonging to distinct families, including Typhlopidae, Gekkonidae, Gymnophthalmidae, Xantusiidae, Teiidae, Scincidae and Lacertidae [12,13]. In recent years, studies focused on facultative parthenogenesis in snakes have produced innovative and field-advancing outcomes, substantially enriching our knowledge and comprehension of this fascinating reproductive phenomenon in these reptiles. While obligate parthenogesis is reported in the brahminy blind snake (*Indotyphlops braminus*) through the mechanism of premeiotic endoreplication, facultative parthenogenesis has been shown in other snakes and can be attributed to terminal fusion automixis. Moreover, it has been observed throughout snake evolution, starting with ancient boas (Boidae), pythons (Pythonidae) and Caenophidia [11,12]. This form of reproduction has been detected in multiple lineages of more modern or ‘advanced’ snakes [11,13,14,15] with several genetically confirmed or anecdotical cases [15,16,17,18,19,20,21,22,23]. Other species involved in similar studies were the western terrestrial garter snake (*Thamnophis elegans*), checkered garter snake (*T. marcianus*), timber rattlesnake (*Crotalus horridus*), Aruba island rattlesnake (*C. unicolour*), arafura file snake (*Acrochordus arafurae*) [24,25], reticulate python (*Malayopython reticulatus*), ball python (*P. regius*) [11] and Burmese python (*Python molurus bivittatus*) [26]. The last-mentioned case was the first one reported in the Pythonidae family, although a more detailed analysis has highlighted some errors of interpretation [15,22]. In another report, a 22-year-old captive Brazilian rainbow boa (*Epicrates cenchria cenchria*) gave birth to four offspring after being housed with a vasectomized male. The male, female and four offspring were genotyped for short tandem repeat (STR) DNA markers. None of the offspring exhibited the specific STR allele found in the possible sire. Furthermore, all the offspring displayed homozygosity at each assessed STR locus, providing evidence for parthenogenetic reproduction [20]. Booth W et al. provided the initial scientifically established evidence of facultative parthenogenesis in a primitive snake from the Boidae family (*Boa constrictor*). This discovery was made possible through the application of microsatellite DNA fingerprinting. The increased homozygosity observed in the offspring compared to the mother implies that the mechanism behind this phenomenon might be terminal fusion automixis. Notably, no male offspring were generated, potentially pointing towards maternal sex chromosome hemizygosity [22]. Whether parthenogens demonstrate reproductive competency is pivotal in understanding the evolutionary and ecological importance of facultative parthenogenesis in wild squamates and other vertebrates. Extensive research on domestic turkeys has recorded the reproductive capability of parthenogens [6,10]. Also, Straube et al. documented reproductive competency in a parthenogenetic whitespotted bamboo shark, where the parthenogen itself produced a parthenogen [12]. In that study, a captive female produced multiple parthenogens. Unexpectedly, out of a collective of nine parthenogens, a solitary specimen exhibited external claspers typically linked to the male gender among chondrichthyans. Upon closer examination through dissection, this particular specimen showed misshapen or missing internal sexual organs. Notably, the existence of claspers in this study raises questions about the previously assumed mechanism of sex determination in this species of shark. In this study, a possible case of parthenogenesis in a ball python (*P. regius*) was analyzed by genotyping five specific microsatellites. The genetic comparisons between mother and embryos demonstrated the parthenogenetic origin of the latter, showing that this reproductive strategy is not rare in this species [11].

## 2. Material and Methods

### 2.1. Animals

A 10-year-old, wild-caught, 1.3 kg female ball python (*P. regius*) laid 4 eggs without ever being in contact with a male. The eggs were promptly removed by the owner and not incubated. After 3 days, an expert veterinarian was asked for a complete check-up of the snake. The snake was born in captivity and acquired at approximately 3 months old; husbandry and diet were considered appropriate for the species [27]. The veterinarian analyzed the egg content, finding residual blood vessels and a small red spot compatible with an early-stage embryo. All the eggs were then submitted to the University of Parma for analysis. Blood (1.3 mL) was collected from the female python by jugular venipuncture and equally divided into two test tubes, one with lithium heparin and one with kEDTA. Genetic analyses were conducted on three embryos, labeled 3068, 3069 and 3144.

### 2.2. DNA Extraction and Amplification

DNA was extracted from the three embryos and from the blood of the mother using the commercial Wizard Genomic DNA extraction kit (Promega, Madison, WI, USA) according to the manufacturer’s instructions. DNA yield and purity were measured on a BioPhotometer (Eppendorf, Hauppauge, NY, USA) and showed the following requirements: yield > 30 ng/μL, 260/280 ratio > 1.7 and 260/230 ratio > 1.8. Before the PCR amplification, all the genomic DNA samples were diluted at 20 ng/μL in nuclease-free water and immediately used. Each amplification was carried out in a 25 μL final volume containing 100 ng of genomic DNA, a 1× PCR buffer, 200 μM of each dNTP, 10 pmol of each primer, and 1 U of GoTaq^®^ G2 Flexi DNA Polymerase (Promega–Madison, Fitchburg, WI, USA).

The thermal conditions were as follows: 98 °C for 30 s, 30 cycles at 98 °C for 10 s, annealing at 64 °C for 30 s and extension at 72 °C for 30 s. A final extension was carried out at 72 °C for 10 min. The amplification products were later verified by electrophoresis on a 2% agarose gel (Bio-Rad, Hercules, CA, USA) in a 0.5X TBE buffer and stained with SYBR^®^green (Lonza Rockland, Inc., Rockland, ME, USA).

### 2.3. Choice of Microsatellites

To verify the genomic identity between mother and embryos, microsatellites previously used in *Python regius* were chosen (Table 1). The amplification products were then purified with ExoSap and sequenced with a Brilliant Dye Terminator 1.1 kit (Applied Biosystems™, Waltham, MA, USA) and a 3730xl DNA Analyzer (Applied Biosystems™, Waltham, MA, USA). In this way, it was possible to count the number of repetitions of the repeated sequences and assign the correct allele.

### 2.4. Statistical Analysis

To statistically support the parthenogenetic event, we calculated the probability of sexual reproduction in the presence of sperm storage. The calculation procedure was carried out according to Booth et al. (2014) [11].

## 3. Results

The genetic material obtained made it possible to genotype all three embryos for the five microsatellites considered. The results obtained are shown in Table 2. For the 15 microsatellite–embryo combinations, homozygosity was observed at all loci.

Considering that the allele frequencies for the selected microsatellites in the population of origin of the studied female are not known, it was not possible to calculate the probability of the parthenogenetic event according to the classical scheme. In this case, we calculated the probability of sexual reproduction in the presence of long-term sperm storage, as reported by Booth et al. (2014) [11]. Results are reported in Table 3.

## 4. Discussion

This study can be considered the fourth report of facultative parthenogenesis (FP) in a captive-bred female ball python (*P. regius*), as previously reported by Booth et al. [11,15]. Parthenogenesis is also documented in oviparous snakes [26], while obligate parthenogenesis is present in only a single lineage of an extant basal scolecophidian snake, the common blind snake (*I. braminus*) [29]. Currently, more than a million ball pythons (*P. regius*) are being kept in captivity and zoos, making them one of the most favored choices among pet snake enthusiasts [30]. Indigenous to Central and Western Africa, these snakes have a typical length range of 0.9–1.8 m (3–6 feet) and can thrive for over 30 years in captivity [30]. Their generally calm temperament enhances their appeal as a sought-after pet choice. Since 1976, Ghana and Togo have been the primary sources, contributing nearly 100% of the specimens that are predominantly exported to the USA and EU. Consequently, ball pythons have emerged as the most extensively traded CITES-listed species originating from Africa, with hundreds of thousands of these creatures being involved in international trade annually [31]. In their natural habitat, royal pythons primarily engage in breeding activities spanning from mid-September to mid-November, aligning with the duration of the rainy season. These pythons follow an oviparous reproductive strategy, with females typically laying an average clutch of six eggs. However, the range of clutch sizes can vary from as few as one egg to as many as eleven [31]. Currently, there is only one study regarding FP in ball pythons [11]. In this study by Booth et al., complete genomic DNA was isolated from the molted skins of living individuals or muscle tissue obtained from deceased ball python embryos. These samples were sourced from three separate and unrelated egg clutches that were generated within three distinct private collections [11]. After mating, some female snakes can store male sperm within their reproductive tracts for extended periods of time, laying fertile eggs after years of reproductive inactivity [32,33,34,35,36]. In a study by Booth et al., a rattlesnake (*Crotalus adamanteus)* was found to hold one of the longest genetically verified instances of long-term sperm storage (LTSS) among vertebrates. In that study, it was captured when it was a young adult. Approximately 60 months later, it gave birth to a substantial and healthy litter consisting of 19 offspring, including 9 males and 10 females [23]. In a more recent study by Levine et al., a female western diamond-backed rattlesnake (*Crotalus atrox*) collected from the wild and kept in isolation since her capture in September 1999 gave birth to two healthy litters, with the intervals between the births being approximately one and six years following her capture. An analysis of the genetic makeup of the 2005 litter revealed the presence of paternal genetic contributions in all offspring [35]. As the female ball python under study was kept without a male since it was sexually immature, this mechanism of internal fertilization was excluded. In the study by Groot et al. [26], a histopathologic examination was performed on seven 24-day-old, 18 cm long embryos for sex determination; based on the presence of ovaries, all offspring were identified as female [15]. Considering that pythons possess sex chromosomes in the form of XX/XY, where XX corresponds to females, the embryos in this case, although of insufficient size for sex determination, would necessarily have been of the female sex [37]. In this study, we analyzed three 2 mm long early-stage snake embryos. The results of this study are in line with those of Booth et al. [15], where all embryos showed homozygosity for the tested microsatellites, despite some being heterozygous in the mother. The presence of only one subset of maternal microsatellites might be interpreted as a sign of terminal fusion automixis, considered to be the developmental mechanism mainly underlying vertebrate FP and characterized by the restoration of diploidy through the fusion or duplication of meiotic products. Among the automictic modes, terminal fusion is the most common mechanism [1,12,35,36], under which the egg nucleus fuses with its second polar body, resulting in highly reduced levels of heterozygosity throughout the genome.

## 5. Conclusions

The case report described in this paper, relating to the microsatellite analyses carried out in a female ball python and in three early-stage embryos, demonstrates with certainty a phenomenon of FP in their origin. As of today, the use of few-day embryos as a source of genetic material to demonstrate parthenogenesis has not been previously documented, presenting new opportunities to utilize this biological material for this purpose. Furthermore, the increasingly frequent identification of cases of FP in *P. regius* demonstrates that this species represents an excellent natural animal model for studying the mechanisms underlying FP.

## Figures and Tables

**Table 1:** Characteristics of the microsatellites used.

Name	All ^a^	Ho ^b^	He ^c^	PIC ^d^	Primers	Reference
MS9	7	0.78	0.79	0.75	5′-CAGTGGGCTTGAGATTGAC-3′	[27]
					5′-CCATTCCTTAAAACACTCTCACTC-3′	
MS5	25	0.91	0.93	0.92	5′-TAGGGTGTCAGTCATTGCTC-3′	[27]
					5′-TGGCATCCAGCAGTCATAG-3′	
MS16	10	0.75	0.82	0.78	5′-GAGTTCTGGTCTTGCTTTCG-3′	[27]
					5′-CAGGTACAACTTTCTCCAAC-3′	
Pmbl-12	4	0.55	0.59	n.a. ^e^	5′-GCCACGTCTAAGGTTGAGC-3′	[28]
					5′-AAAGCAGGTCTCTGTTGGG-3′	
KE955519	8	0.75	0.72	0.68	5′-ATTTTAGCTGCAGGCTGTGG-3′	[27]
					5′-TCTGCTAGGGCAAAACTGGG-3′	

^a^ number of alleles; ^b^ observed heterozygosity; ^c^ expected heterozygosity; ^d^ polymorphism information content; ^e^ not available.

**Table 2:** Genotypes of the mother and offspring at five microsatellite loci.

	MS9	MS5	MS16	Pmbl-12	KE9555519
Mother	164/168	346/362	350/366	480/480	346/348
3068	164/164	362/362	366/366	480/480	346/346
3069	168/168	346/346	366/366	480/480	348/348
3144	164/164	362/362	366/366	480/480	346/346

**Table 3:** Probability of sexual reproduction.

	Number of Offspring	Number of Maternally Homozygous Loci	Number of Maternally Heterozygous Loci	Probability of Long-Term Sperm Storage (per Individual)	Probability of Long-Term Sperm Storage (per Clunch)
Mother	3	1	4	0.001953	7.45 × 10^−9^

## Data Availability

Not applicable.
